# The Connection Between Sleep Problems and Emotional and Behavioural Difficulties in Autistic Children: A Network Analysis

**DOI:** 10.1007/s10803-024-06298-2

**Published:** 2024-03-25

**Authors:** Lucy Sommers, Nicole Papadopoulos, Matthew Fuller-Tyszkiewicz, Emma Sciberras, Jane McGillivray, Patricia Howlin, Nicole Rinehart

**Affiliations:** 1 School of Psychology, Faculty of Health, Deakin University, 1 Gheringhap Street, Geelong, VIC 3220 Australia; 2 Monash Krongold Clinic, Faculty of Education, Monash University, 19 Ancora Imparo Way, Clayton, VIC 3800 Australia; 3 School of Educational Psychology and Counselling, Faculty of Education, Monash University, 19 Ancora Imparo Way, Clayton, VIC 3800 Australia; 4 Murdoch Children’s Research Institute, 50 Flemington Road, Parkville, VIC 3052 Australia; 5 Department of Paediatrics, University of Melbourne, Grattan Street, Parkville, VIC 3010 Australia; 6 Institute of Psychiatry, Psychology and Neuroscience, King’s College London, 16 De Crespigny Park, London, SE5 8AF UK; 7 School of Psychology, Deakin University, 221 Burwood Hwy, Burwood, VIC 3125 Australia

**Keywords:** Children, Autism, Autism Spectrum Disorder, Emotional and Behavioural Difficulties, Sleep Problem, Anxiety, Depression, Network Analysis

## Abstract

The interactions between sleep problems, autism symptoms and emotional and behavioural difficulties were explored using network analysis in 240 autistic children (mean age: 8.8 years, range 5–13 years) with moderate to severe sleep problems. Findings revealed a highly connected and interpretable network, with three separate clusters identified of the modelled variables. Depression, anxiety and behavioural difficulties were the most central variables of the network. Depression, anxiety and restricted repetitive and stereotyped patterns behaviours (RRBs) were the strongest bridging variables in the network model, transmitting activation both within and between other symptom clusters. The results highlight that depression and anxiety were highly connected symptoms within the network, suggesting support in these areas could be helpful, as well as future research.

**Supplementary Information:**

The online version contains supplementary material available at 10.1007/s10803-024-06298-2.

## Key Points

• Studies have found that autistic children experience a high rate of sleep problems, which worsen autism symptoms and increase emotional and behavioural difficulties.

• Network analysis offers an advanced statistical approach to explore complex interactional patterns of co-occurring symptoms and identify possible intervention targets, ultimately improving supports for autistic children and their families.

• The network model produced, was highly connected and interpretable, with three distinct symptom clusters identified.

• The variables of depression, anxiety and behavioural difficulties were the most central variables of the network model.

• The variables of depression, anxiety and RRBs emerged as the strongest bridging variables within the network model and could be the focus of supports for children and their families.

**Supplementary Information:**

The online version contains supplementary material available at 10.1007/s10803-024-06298-2.

Autism spectrum disorder (hereafter ‘autism’), is a neurodevelopmental condition, characterised by deficits in social (e.g., abnormal social approach), communication (e.g., eye contact and body language) and restricted repetitive patterns of behaviour (RRBs), interests or activities (e.g., motor stereotypies, fixated interests, sensory sensitivities) (American Psychiatric Association [APA], 2013). Autism is estimated to affect at least 1% of the population worldwide (APA, 2013) and is associated with a high prevalence of co-occurring conditions. In particular, 40–93% of autistic children are estimated to experience sleep problems (Carmassi et al., 2019), including bedtime resistance, difficulty initiating and maintaining sleep, reduced sleep duration, early morning awakenings and, excessive daytime sleepiness (Díaz-Román et al., 2018; Krakowiak et al., 2008; Liu et al., 2006; Richdale, 1999). Sleep anxiety is also common, resulting in a parent being present to fall asleep, as well as rigid adherence to learned sleep associations and/or bedtime resistance (Chen et al., 2021; Hoffman et al., 2006; Owens et al., 2000).

While sleep problems also present in non-autistic children, prevalence rates in autistic children are significantly higher (Gringras, 2014; May et al., 2015) and more enduring (Sivertsen et al., 2012). Sleep problems in autistic children are correlated with heightened rates of emotional and behavioural difficulties (Lindor et al., 2019; Schreck, 2021) including tantrums, oppositional behaviour, physical aggression, irritability, inattention, hyperactivity, self-stimulatory behaviours, depression, and anxiety (Goldman et al., 2009, 2011; Mayes & Calhoun, 2009; Mazurek & Petroski, 2015; Park et al., 2012; Sikora et al., 2012). Sleep problems experienced at a moderate to severe level in autistic children, correlate with more internalising and externalising behavioural difficulties than children with mild to moderate sleep problems (Sikora et al., 2012). In addition, the severity of emotional and behavioural difficulties (i.e., anxiety, depression, withdrawal, somatic complaints, socialisation problems, rule-breaking, and aggression) for autistic children with milder sleep problems is directly correlated with severity of autism symptoms, while autistic children with moderate to severe sleep problems experience emotional and behavioural difficulties at clinically high levels, irrespective of autism symptom severity (Lindor et al., 2019).

Autistic children typically experience multiple, and concurrent sleep problems (Liu et al., 2006) with severity worsening over time, compared to non-autistic peers (Hodge et al., 2014; Verhoeff et al., 2018). Literature has demonstrated a significant relationship between sleep problems and sensory processing difficulties, which are encompassed within RRBs (Mazurek & Petroski, 2015; Tzischinsky et al., 2018). Specifically, sensory hypersensitivity towards touch was found to interfere with sleep (Tzischinsky et al., 2018). In addition, sleep problems are correlated with an increase in autism symptoms (Hollway & Aman, 2011; Veatch et al., 2017), including self-injurious, compulsive (Goldman et al., 2011) and repetitive behaviours (Park et al., 2012). Shorter sleep duration in autistic children has been found to be strongly correlated with RRBs, severe social impairment and failure to develop peer relationships (Veatch et al., 2017), in addition to more severe autism symptoms (Schreck et al., 2004). Tudor et al. (2012) found night waking was correlated with communication difficulties and a predictor of social interaction difficulties. In addition, Tudor et al. (2012) also found parasomnias were significantly related to stereotyped behaviours, communication and overall autism severity. Furthermore, children who awake screaming in the night show higher rates of stereotypic behaviours and greater communication abnormalities (Schreck et al., 2004).

In an area where co-occurrence is the norm rather than the exception, network analysis offers an advanced statistical approach to explore complex interactional patterns of co-occurring symptoms and identify possible intervention targets, ultimately improving supports for autistic children and their families. Network analysis maps partial correlations among variable pairs, controlling for shared associations with other variables (or ‘nodes’ in network analysis terminology) in the overall network. In this way, spurious associations driven by third variables are minimised, enabling assessment of which variables within a network are most influential. This assessment of the influence (or ‘centrality’ in network analysis terminology) of a variable in the network is ascertained by aggregating all of the connections that a variable has with others in the network. Centrality may take the form of either strength of direct connections to other variables (this metric is labelled ‘strength’, and is perhaps the most common and robust metric used for evaluating variables in a network) (Robinaugh et al., 2020) or ability to be a connecting variable between other variables that are not directly connected (this metric is labelled ‘betweenness’). In either case, the influence of a variable is detected through the aggregated strength of associations it has with other variables in the network.

In addition to focusing on individual, influential variables in the network, network analysis also enables the grouping of variables through detection of clusters of symptoms/nodes which form from the relationships between symptoms in the network (Golino & Epskamp, 2017). Researchers within the field of clinical psychology have also endeavoured to utilise network analysis to further understand the presentation of co-occurring conditions, through the identification of bridging symptoms (Jones, Ma, & McNally, 2021). Broadly, bridging symptoms are variables that are shown to relate to multiple clusters of variables (in this case, the clusters would represent distinct psychological conditions) (Cramer et al., 2010). Thus, a variable’s strength value indicates that it is connected to other variables in the model, with bridging metrics specifically designed to identify variables that are related to two or more clusters of variables.

These bridging variables are theorised as potential sources of co-occurrence. As such, bridging variables provide a unique opportunity to deliver support in one area, which simultaneously has a flow-on effect in other areas within the network (Borsboom & Cramer, 2013). Until recently, network analysis within the field of autism research, has primarily used genetic, neuroimaging and metabolic data. In recent years, the application of network analysis has expanded to a behavioural and symptom level (Anderson et al., 2015; Ruzzano et al., 2015). Recently, Montazeri et al. (2020) utilised network analysis in a sample of 118 autistic children aged 9–13 years, examining the interaction between autism symptoms, anxiety, depression, and obsessive-compulsive disorder. Based on their findings and recommendation to target anxiety and insomnia in the treatment of depression in autistic children, we conducted further exploration of the interactional pattern of co-occurring conditions. In addition to using a larger sample of autistic children, we built on the work of Montazeri et al. (2020) by using the Child Sleep Habits Questionnaire - Autism (CSHQ-Autism), a validated measure of sleep problems in autistic children (Katz et al., 2018), opposed to an individual sleep item, to further expand the literature within these symptom domains.

The aim of the present study was to employ network analysis to investigate the interconnections between autism symptoms, sleep problems and emotional and behavioural difficulties in autistic children with moderate to severe sleep problems. The three questions addressed were:

1. In a network model, which symptoms cluster together (e.g., sleep vs. autism symptoms vs. emotional and behavioural difficulties)?
2. Which variables are most important in the network structure, as assessed by node strength?
3. Which variables are identified as bridging symptoms within the network model?

## Method

### Design and Setting

 This study is based on baseline data from the Sleeping Sound Autism study, a randomised controlled trial (RCT) examining a behavioural sleep intervention for autistic children (Papadopoulos et al., 2019). This project was granted ethics approval by the Royal Children’s Hospital (No 36154) and Deakin University (No 2017-130) Human Research Ethics Committees.

### Recruitment and Procedure

Participants were recruited through Paediatric clinics in Victoria, Australia via (a) a letter of invitation to participate in the study, sent to children seen in the last 12 months, and (b) a telephone call to families who had registered interest in the study (Papadopoulos et al., 2019). Families who met eligibility criteria were sent an information pack, consent form, and a parent survey.

### Participants

Participants were 240 autistic children (mean age 8.8 yrs, SD 2.1; range 5–13) with moderate to severe sleep problems. Inclusion criteria were: (i) a multidisciplinary diagnosis of Autistic Disorder or Asperger’s Disorders, based on the Diagnostic and Statistical Manual of Mental Disorders, 4th edition (DSM-IV) or DSM-5 Autism Spectrum Disorder diagnosis, as indicated by their paediatrician and confirmed in a clinical report; (ii) aged 5–12 years or 13 years and attending primary school at the time of recruitment; (iii) a score of ≥11 on the Social Communication Questionnaire – Lifetime form (Rutter et al., 2003); (iv) a moderate or severe sleep problem persisting for ≥ 4 weeks, ascertained by parent/caregiver report (Sung et al., 2008; Thomas et al., 2018); (v) parents who indicated a moderate or severe problem were then asked additional questions to determine whether the child met the diagnostic criteria for at least one of the following parent/caregiver reported sleep problems as defined by the International Classification of Sleep Disorders – Third Edition criteria for chronic insomnia (including difficulty initiating and maintaining sleep, early morning waking, bedtime resistance, sleep-onset association, sleep anxiety) and/or delayed sleep-wake phase (American Academy of Sleep Medicine, 2014). Participants taking melatonin as well as any other medication (e.g., SSRI) were included if they met all other eligibility criteria.

Exclusion criteria included: (i) parent/caregiver reported intellectual disability diagnosis; (ii) insufficient English to provide informed consent and complete research measures (Papadopoulos et al., 2019); (iii) co-occurring medical condition in child that disturbs regular sleep (e.g. blindness, epilepsy, traumatic brain injury, and/or neuropsychiatric disorder such as Tourette’s syndrome) or genetic condition related to intellectual impairment (e.g. Fragile X disorder); and (iv) suspected Obstructive Sleep Apnoea (OSA). If the parent/ caregiver endorsed three Sleep Disordered Breathing items on the Children’s Sleep Habits Questionnaire (Owens et al., 2000) during screening, the child was referred to the study paediatrician for diagnostic clarification; parents were offered the opportunity to participate at a later time if the child was found to not have OSA (Papadopoulos et al., 2019).

### Measures

*Autism symptoms* were measured by the parent-reported Social Communication Questionnaire (SCQ) (Berument et al., 1999; Rutter et al., 2003). Two versions of the SCQ were utilised (Papadopoulos et al., 2019). The SCQ-Lifetime (SCQ-L), administered during screening procedures, confirmed whether children met the clinical cut-off (score ≥11) for study eligibility (Corsello et al., 2007); the SCQ-Current (SCQ-C) was administered at baseline to assess the presence of behaviours during the past three months. Of the 40 items, 13 assess communication functioning, 15 reciprocal social interactions and 8 restricted, repetitive and stereotyped patterns of behaviour. Total scores range from 0 to 39 with higher scores representing greater impairment. The SCQ-L and SCQ-C have good internal consistency, α = 0.82 and α = 0.79 respectively, good construct validity and good convergent validity (Ung et al., 2016).

*Emotional and Behavioural Difficulties* were assessed using the Developmental Behaviour Checklist – Primary Carer Version (DBC-P) (Einfeld & Tonge, 1995), a 96-item questionnaire based on the past six months (each item rated from 0 [*no known problem*] to 2 [*very/often true])*. This study utilised the disruptive/antisocial subscale (27 items) to measure behavioural difficulties, and the anxiety (9 items), depression (10 items) and hyperactivity (6 items) subscales. The DBC has satisfactory to good inter-rater, test-retest reliability and concurrent validity particularly in children/ adolescents with intellectual disabilities (Einfeld & Tonge, 1996, 2002). Internal consistency levels range from excellent to satisfactory: total score (α = 0.94), disruptive and antisocial behaviour subscale (α = 0.90), hyperactivity (α = 0.80), anxiety (α = 0.70) and depression (α = 0.66).

*Sleep Problems* were assessed with the parent-reported Child Sleep Habits Questionnaire (CSHQ). This study utilised the Katz and colleagues (Katz et al., 2018) modified scoring of the CSHQ. Derived from the original 33-item CSHQ (Owens et al., 2000), the modified measure excluded ten items following the examination of distribution and factor loading, leaving a 23-item measure, useful for the screening of sleep problems in autistic children (CSHQ-Autism) (Katz et al., 2018). Each item is rated on a 3-point scale from 1 = *rarely* to 3 = *usually*, with higher scores indicating greater sleep problems. The four subscales utilised in this study were sleep initiation and duration (6 items), sleep anxiety/co-sleeping (5 items), night waking/parasomnias (6 items) and daytime alertness (6 items). Internal consistency levels in the current study ranged from good to acceptable: sleep anxiety/co-sleeping (α = 0.83), daytime alertness (α = 0.82), total score (α = 0.76), night waking/parasomnias (α = 0.70) and sleep initiation and duration (α = 0.63).

*Additional parent-reported information* was collected including child age, sex, medication use, co-occurring conditions and family socioeconomic status (SES). Medication use was ascertained by asking *“What type of medication is your child currently taking to assist with learning, behaviour, emotional or sleep difficulties?”* with responses categorised into five groups (ADHD, antipsychotic, antidepressant, sleep and bipolar). Co-occurring conditions were assessed by asking *“In addition to Autism, has your child been diagnosed with or treated for any of the following by a health professional?”* with responses categorised into three groups (ADHD, internalising i.e., anxiety & depression, externalising i.e., conduct & oppositional defiant disorder). SES was assessed using family residential postcode and the Australian Bureau of Statistics Socio-Economic Indexes for Areas (SEIFA), Australia, 2016-Index of Relative Socio-Economic Disadvantage (Australian Bureau of Statistics, 2018b). This score is standardised with a mean of 1000 with lower scores indicative of greater socio-economic disadvantage (Australian Bureau of Statistics, 2018a).

### Data Analysis

Network analyses were performed and visualized in R (r-project.org), using the packages qgraph (Epskamp et al., 2012), igraph (Csardi & Nepusz, 2006), exploratory graph analysis (EGA) (Golino, 2018) and network tools (Jones, 2020). Adaptive lasso was used for network estimation. In the network visualisation, each node represents a subscale from the DBC, SCQ-C and CSHQ-Autism.

Several statistics were used to characterise the network of sleep problems, autism symptoms and emotional and behavioural difficulties. Exploratory graph analysis (Golino & Epskamp, 2017) detected the number of clusters in the data and which symptoms belonged to each cluster. Centrality and bridge influence (expected influence [EI1]) metrics were used to determine the importance of individual symptoms within the network (Robinaugh et al., 2016). The centrality measure of *strength* was used to provide an aggregate assessment and refers to how strongly a variable node is directly connected to all other variables in the network. *Bridging influence* represents variables in the network that connect two different symptoms and possibly act as a pathway between co-occurring symptoms and subsequently represent a potential avenue for support. In line with previous research (Forrest et al., 2019), this study specifically focused on *bridge EI1* which measures the node’s influence with its immediate neighbours in the network (Robinaugh et al., 2016). Bridge EI1 values do not consider which cluster variables may belong and may thus reflect some combination of within- and between-cluster connections for a given variable. In the Results and Discussion sections, we attempt to explicitly delineate connections that are within- vs. between-cluster when describing bridge EI1 results. This is based on the notion that targeting a variable with between-cluster connections may be a means to intervene more efficiently on several clusters of variables simultaneously (Montazeri et al., 2020).

The correlation stability coefficients (*CS*-coefficient) were used to evaluate the stability and interpretability of the obtained network (Epskamp et al., 2017). The CS-coefficient represents the proportion of participants that can be dropped from the sample while maintaining a correlation of at least 0.70 with observed network coefficients (strength) for the full sample (Epskamp et al., 2017). Correlation between the results for the overall sample and increasingly small subsets of the sample indicate potential uniformity in results for the overall sample, through which stability of one’s results may be gauged. A network is deemed sufficiently stable if the CS-coefficient is greater than 0.25 (i.e., correlation of at least 0.7 between subset and full sample results when dropping 25% of the overall sample), though ideally values greater than 0.50 are preferred (Epskamp et al., 2018).

## Results

### Sample Characteristics

Sample characteristics are reported in Table 1 and information on child sleep problems, emotional and behavioural difficulties, and autism symptoms in Table 2. Within the sample, 65% were male; the mean current SCQ score was 14.35 (*SD* = 5.75, range: 3–29); 48% were taking sleep medication; 37% were taking medication for ADHD; 47% had a parent-reported diagnosis of an internalising condition (e.g., anxiety or depression) and 40% a diagnosis of ADHD. During screening, child sleep problems reported by parents included: chronic insomnia (99%), delayed sleep-wake phase (12%), sleep anxiety (56%), somnambulism (10%), sleep terrors (16%), nightmares (5%).

**Table 1:** Sample characteristics

Characteristics	*n* (%)
*Child*	
Age years: *M (SD), range*	8.80 (2.12) *5–13*
Male	155 (65.35%)
SCQ-Lifetime: *M (SD), range*	20.12 (5.48) *11–34*
Medication Use	
ADHD	89 (37.08)
Antipsychotic	19 (7.92)
Antidepressant	40 (16.67)
Sleep	115 (47.92)
Bipolar	0
Co-occurring conditions	
ADHD	95 (39.58)
Internalising conditions	112 (46.67)
Externalising condition	23 (9.58)
Co-occurring internalising & externalising conditions	18 (7.50)
Family	
Family socio-economic status *M (SD)*, range	1032.86 (55.28) 795–1117

**Table 2:** Descriptive statistics of study variables

Variable	M (SD)	Range
*SCQ-C*		
Total score	14.35 (5.75)	3–29
Reciprocal social interactions	3.98 (2.86)	0–13
Communication	5.06 (2.28)	0–12
Restricted, repetitive & stereotyped patterns of behaviour	3.90 (2.16)	0–8
*DBC*		
Total score	64.13 (24.57)	8-133
Behavioural difficulties	23.01 (9.97)	2–51
Anxiety	7.95 (3.70)	0–18
Hyperactivity	7.16 (2.95)	0–12
Depression	7.34 (3.23)	0–16
*CSHQ-Autism*		
Total Score	43.52 (7.35)	26–63
Sleep initiation and duration	12.03 (2.50)	6–17
Sleep anxiety/co-sleeping	8.71 (3.24)	5–15
Night waking/parasomnias	11.18 (2.83)	6–18
Daytime alertness	11.60 (3.36)	6–18

SCQ-C: Social Communication Questionnaire-C; DBC: Developmental Behaviour Checklist; CSHQ-Autism: Child Sleep Habits Questionnaire-Autism (*N* = 240)

### Stability of Network Analysis

Network stability analysis revealed the CS-coefficient for network edges (0.75), node strength (0.52) and bridge strength (0.75) were greater than the recommended cut-off of 0.25. (Epskamp et al., 2017) and are subsequently interpretable.

### Network Structure and Clustering of Symptoms

**Fig. 1:**
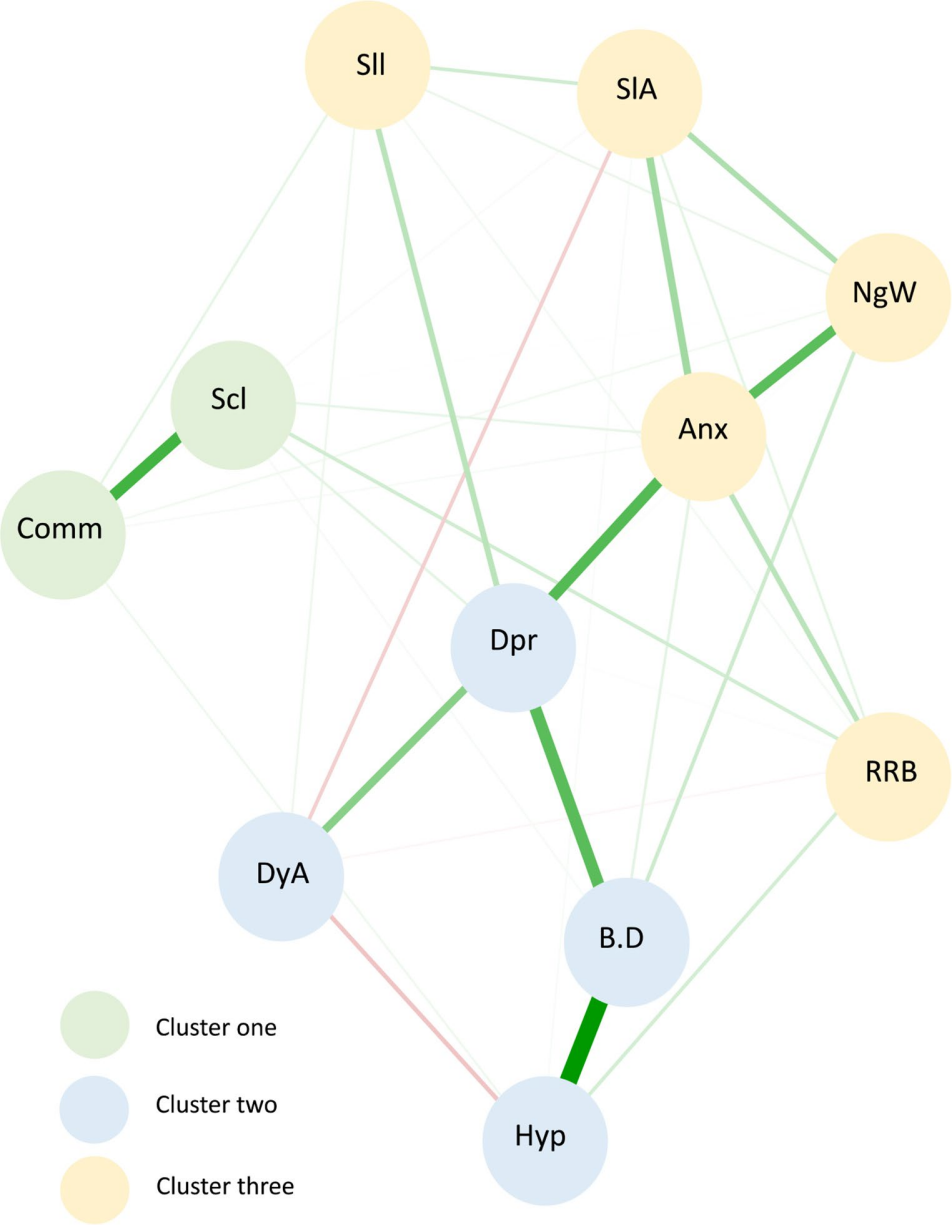
Network model of sleep problems, autism symptoms and emotional and behavioural difficulties. *Note.* The three clusters identified within the network model are reflected by numerical values ‘1, 2, 3’ and (cluster one = green; cluster two = blue; cluster three = yellow). Autism symptom label descriptions: Scl = Reciprocal social interactions; Cmm = Communication; RRBs = Restricted, repetitive & stereotypes patterns of behaviour. Sleep problem label descriptions: NgW = Night waking/parasomnias; SlA = Sleep anxiety/co-sleeping; Sll = Sleep initiation & duration; DyA = Daytime alertness. Emotional and behavioural difficulty label descriptions: B.D = Behavioural difficulties; Anx = Anxiety; Hyp = Hyperactivity; Dpr = Depression. Tie strength is indicated by line thickness between nodes with thicker lines representing stronger ties. The lines between each node represent either a positive correlation displayed as green or a negative correlation displayed as red

The network (see Fig. 1) identified three separate clusters for the 11 modelled variables. Of note, within each cluster formed, symptoms do not exclusively represent symptom domains of sleep, autism and emotional and behavioural difficulties, but rather a combination across the three domains. Cluster one (green) included autism symptoms: communication and social interaction. Cluster two (blue) comprised of emotional and behavioural difficulties: depression, behavioural difficulties, and hyperactivity; and sleep problem: daytime alertness. Cluster three (yellow) consisted of sleep problems: night waking/parasomnias, sleep anxiety/co-sleeping and sleep initiation and duration; emotional and behavioural difficulty: anxiety and autism symptom: RRBs.  Based on the width of lines (edges) connecting variables within the network, strong relationships exist (after controlling for other symptoms within the cluster and overall model) for: (1) social interaction and communication (cluster one), (2) behavioural difficulties and hyperactivity (cluster two), (3) behavioural difficulties and depression (cluster two), (4) daytime alertness and depression (cluster two), (5) anxiety and night waking/parasomnias (cluster three), depression (cluster two) and sleep anxiety/co-sleeping (cluster three). Partial correlations between variables are outlined in Supplementary Table 1.

### Centrality

**Fig. 2:**
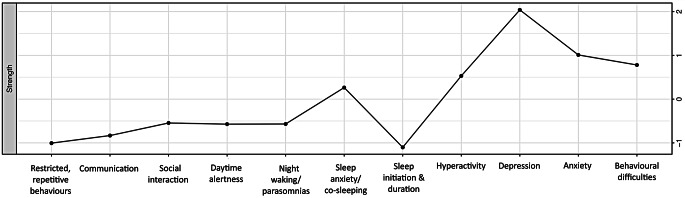
Centrality measures of all variables within the network. Figure showing the centrality measure (i.e., node strength) of all symptoms within the network. *Note.* Higher numbers indicate the item is more central to the network. The nodes with the highest ‘node strength’ (i.e., depression, anxiety and behavioural difficulties) reflect nodes with the most and strongest connections

The identification of central variables within the network model is depicted in Fig. 2 with centrality values for each node outlined in Supplementary Table 2. Stability analysis (see Supplementary Fig. 1) revealed the correlation stability (CS) coefficients indicate node strength (*C S*(cor = 0.7) = 0.517) reached the required cut-off of 0.5 indicating the metric was stable. Node strength illustrates that depression, anxiety and behavioural difficulties are the most strongly connected nodes in this network.

### Node Bridge Influence

**Fig. 3:**
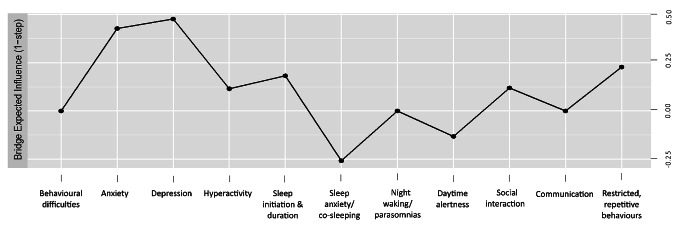
Plot denoting the bridge one-step expected influence metric for the graphical LASSO network. *Note.* Higher numbers indicate that the item is more central to the network

Bridging symptoms within the network are illustrated in Fig. 3 and depicted by bridge one-step expected influence (EI1) values. Depression, anxiety and RRBs had the highest bridge EI1, with connections both within and between clusters. Depression had strongest within-cluster connections to behavioural difficulties and daytime alertness in cluster two, and strongest between-cluster connections to anxiety and sleep initiation and duration (cluster three), and small connections to cluster one variables. Anxiety had the strongest within-cluster connections to night waking/parasomnias, sleep anxiety/co-sleeping and RRBs; and the strongest between-cluster connection to depression (cluster two), and small connections to cluster one variables. RRBs also demonstrated connections across all three clusters, with the strongest within-cluster connection to anxiety; and the strongest between-cluster connections with social interaction (cluster one), and hyperactivity (cluster two).

## Discussion

We conducted a network analysis exploring the interactional pattern between autism symptoms, sleep problems and emotional and behavioural difficulties in autistic children with moderate to severe sleep problems. Findings revealed an interpretable, highly connected network, providing an alternative method of conceptualising the interactional pattern between symptoms. Within the network, three symptom clusters were produced with depression, anxiety and behavioural difficulties most central and influential in the network. Depression and anxiety, in addition to RRBs, were identified as bridging variables, which, if disrupted are most likely to reduce connectivity between symptoms and subsequent activation within the network.

Within cluster one, two autism symptoms were grouped: communication and social interaction. This finding is in line with the current DSM-5, autism criteria (APA, 2013), where communication and social interaction are merged into a single criterion. In addition, a within-cluster relationship was evidenced by a strong partial correlation. Similarly, the network model produced by Montazeri et al. (2020) found communication and reciprocal social interaction nodes exhibited a strong partial correlation, with RRBs remaining, however, on the periphery of the autism cluster. In contrast to previous research where communication difficulties have been linked with sleep onset delay, sleep duration, parasomnias, overall sleep disturbance (Tudor et al., 2012) and environment sensitivities (noise, light etc.)(Schreck et al., 2004), such associations were not evident in this study’s findings. In addition, previous regression analyses indicating a strong association between severe social impairment (i.e., failure to develop peer relationships) and shorter sleep duration (Veatch et al., 2017), were not reflected in the current study’s symptom clustering.

Within cluster two, four variables were grouped: three emotional and behavioural difficulties (depression, behavioural difficulties, hyperactivity) and sleep problem, (daytime alertness). The finding that daytime alertness is encompassed within a profile that also includes behavioural difficulties and hyperactivity is in line with studies exploring how children exhibit signs of tiredness (Fallone et al., 2002; Owens, 2007; Roussis et al., 2021). Specifically, prior research has illustrated that unlike adults, who typically exhibit tiredness through responses such as yawning and expressing fatigue, children experience behavioural manifestations including irritability, emotional lability, low frustration tolerance, aggression, impulsivity (Fallone et al., 2002) and inattention (Roussis et al., 2021). Furthermore, the strong partial correlation maintained between behavioural difficulties and hyperactivity is consistent with clinical research identifying the connection between hyperactivity and broader behavioural difficulties (Giannotta & Rydell, 2016). The strong partial correlation between depression and daytime alertness aligns with the diagnostic symptoms of depression (APA, 2013) and past research in non-autistic children (Calhoun et al., 2011).

Within cluster three, five variables were grouped: three sleep problems (night waking/parasomnias, sleep initiation and duration, sleep anxiety/co-sleeping) autism symptom (RRBs); emotional and behavioural difficulty (anxiety). The clustering of these five variables aligns with previous findings in this field. Specifically, Schreck et al. (2004) and Veatch et al. (2017) reported a correlation between RRB and sleep duration, and Park et al. (2012) found RRBs were associated with bedtime resistance and insomnia. Further, increased levels of repetitive behaviour have been found to be correlated with higher anxiety in autism (Rodgers et al., 2012) and parasomnias (Tudor et al., 2012). The clustering of sleep problems, night waking/parasomnias, sleep initiation and duration and sleep anxiety/co-sleeping with anxiety in this study, has been explored in other studies with strong evidence to support the link between these variables. Most recently, a meta-analysis by Han et al. (2022) found internalising symptoms such as anxiety, yielded the strongest association with sleep problems. Similarly, Schreck (2021) found sleep quality and quantity were both predictive of daytime anxiety. These recent studies build on previous literature similarly linking sleep problems with anxiety (Malow et al., 2006; Mayes & Calhoun, 2009; Park et al., 2012). Williams et al. (2015) found sleep onset delay, sleep duration and sleep anxiety were significantly correlated with anxiety in autistic children. Using path analysis, Mazurek and Petroski (2015) similarly found that autistic children with co-occurring anxiety were predisposed to sleep problems, including night waking, sleep initiation difficulties, sleep duration, and sleep anxiety. This research further supports Mazurek and Petroski’s (2015) postulation that autistic children with co-occurring anxiety and sensory over-responsivity (RRBs) may be particularly vulnerable to sleep problems. The formation of this cluster highlights the interconnectedness of these variables, suggesting the co-occurrence of anxiety and RRBs might contribute to sleep problems in autistic children. As a consequence, arousal regulation difficulties may subsequently hinder a child’s ability to fall asleep. Moreover, these children might exhibit heightened sensitivity to environmental stimuli, which could further disrupt their ability to initiate and maintain sleep.

In this study, the separation of depression and anxiety into distinct clusters (clusters two and three respectively) was notable. However, despite this separation, a between-cluster relationship was maintained between these internalising conditions, as evidenced by the strong partial correlation. This finding highlights both the unique characteristics and the interconnected nature of these conditions within autistic children. The distinct placement of depression and anxiety in separate clusters suggests that when either condition is experienced by an autistic child, the specific symptoms experienced may differ. This implies varying symptom profiles associated with depression and anxiety within this population. Nevertheless, despite forming in different clusters, the strong partial correlation indicates common factors between depression and anxiety in autistic children, meaning that although they are distinct entities within the network, they still influence each other. This could signify shared underlying pathways or certain commonalities contributing to both conditions, despite expressing themselves differently within the network. The varying symptom profiles among autistic children with depression and anxiety highlight the importance of individualised care. Specifically, treatment plans should be personalised, considering the unique needs of the child and the challenges they experience to maximise the effectiveness of interventions.

In contrast to previous network studies (Montazeri et al., 2020), the formation of the three clusters, did not exclusively align with domains of autism, sleep problems, and emotional and behavioural difficulties. Understanding the formation of these clusters and how they differ from traditional approaches, offers a more comprehensive understanding of co-occurring symptoms autistic children experience, ultimately aiding in better outcomes by addressing the complex relationships among various symptoms.

In addition to identifying clusters within the network model, we investigated which variables were most central to the network. Depression, anxiety, and behavioural difficulties emerged as most central, with the strongest connections to other variables, either through the number, and/or strength of connections. Specifically, depression was directly connected to six other variables in the network and maintained the strongest connection with behavioural difficulties, anxiety, and daytime alertness. Similarly, anxiety, was directly connected to seven other variables in the network, maintaining the strongest connections with depression, night waking/parasomnias and sleep anxiety/co-sleeping. Behavioural difficulties, while still connected to five other variables in the network, maintained a very strong connection with hyperactivity and depression.

Emphasis on strength metrics further informs future interventions. Our results suggest depression, anxiety, and behavioural difficulties as plausible candidates. Current treatment interventions for autistic children with sleep problems typically focus on behavioural sleep strategies (Bruni et al., 2018; Cortese et al., 2020). While effective (Pattison et al., 2022), the findings of this network study highlight the need and potential benefit of broadening the current intervention model to include and potentially preface the treatment of co-occurring conditions (i.e., anxiety and depression) this population of children experience. By focusing treatment on a child’s anxiety, a flow-on effect may result, influencing other symptoms within the same cluster (i.e., RRBs, night waking, sleep initiation and duration, and sleep anxiety). Similarly, addressing the depressive symptoms a child is experiencing, may help resolve other symptoms in the cluster (i.e., behavioural difficulties, daytime alertness, and hyperactivity). As it stands, co-occurring conditions such as anxiety can have a significant impact on treatment adherence (Santana & Fontenelle, 2011), and as such adopting a transdiagnostic approach to intervention delivery may further improve treatment outcomes for autistic children.

Further to depression and anxiety being the most central variables in the network, they also exhibited the highest bridging values in the network, with strong between-cluster connections. In addition, RRBs and sleep initiation and duration were identified as variables with the next highest bridging values within the network. Both variables had several connections outside of their clusters, suggesting the bridging values were not entirely driven by their within-cluster connections. Sleep initiation and duration had their strongest between-cluster connection with depressive symptoms, while RRBs had their strongest between-cluster connection with social interactions. These findings suggest that anxiety or sleep initiation may be good variables for targeted support to subsequently alter cluster two variables (e.g., dep), whereas RRBs may be the most plausible bridging variable candidate from tested variables to help reduce cluster one symptoms (e.g., social interaction).

Network theory suggests treatments focused on core maintaining symptoms should have maximal effect in decreasing other symptoms within a psychopathology network (Borsboom & Cramer, 2013). This network identified depression, anxiety, RRBs and sleep initiation and duration as variables that have connections to multiple clusters. In terms of prioritising sources of interventions, these bridging variables are attractive candidates for intervention because improvement in them may have flow-on effects, simultaneously deactivating and reducing the overall network connectivity.

Limitations of this study included: (i) The use of cross-sectional data means we are unable to discern the directionality of the associations found in this network (Contreras et al., 2019). Future longitudinal studies would improve predictions about symptoms that play a role in the onset and maintenance of sleep difficulties; (ii) Although the sample size was relatively large for a clinical study, network models estimate a very large number of parameters, and hence much larger samples are required to draw firm conclusions. Encouragingly, stability analyses indicate the likely generalisability of these findings in similar samples. In addition, based on evidence that the types of sleep problems change as autistic children go through puberty (Goldman et al., 2012) it would be important to replicate this study with larger samples, enabling two separate network analyses based on age; (iii) The parent report measures adopted to assess children’s behaviours are subject to inherent biases, with parents potentially over or underreporting the severity of their children’s difficulties. However, the measures utilised in this study are widely used within the field and well-validated; (iv) The depression subscale from the DBC included two items relating to sleep. While this conforms with DSM-5 criteria for depression, the presence of sleep items within the subscale may have led to circularity (i.e., poor sleep predicts poor sleep); (v) At the time of development and participant recruitment for this study, no cut-off score was available to assess sleep problem severity when using the CSHQ-Autism questionnaire. Instead, assessment was based on a parent-reported, moderate or severe sleep problem, persisting for ≥ 4 weeks, an approach previously applied by (Sung et al., 2008; Thomas et al., 2018). Recently, Shui et al. (2021) derived a cut-off score of 35 for the CSHQ-Autism total score in order to identify sleep problems in autistic children aged between 2 and 17 years. This raises concerns about the measurement of sleep problems used in the present study as the reported range (26–63) falls below the cut-off recommended by Shui et al. (2021). While the current sample still represents a sample of autistic children with parent-reported moderate to severe sleep problems, future research would benefit from incorporating the Shui et al. (2021) cut-off score into the study design; (vi) While we did not have a large enough sample size in this study, future research would benefit from conducting a network analysis, utilising individual items within scales (Silk et al., 2019), rather than subscales, to fully examine the relationships present and identify appropriate support pathways; (vii) The generalisability of the findings is limited by the exclusion of autistic children with an intellectual disability and autistic children from non-English speaking backgrounds, with applicability to these populations unknown. Despite these limitations, to our knowledge, this is the first study to use network analysis to examine the cross-associations between emotional and behavioural difficulties in autistic children with moderate to severe sleep problems.

## Conclusion

This study of autistic children highlights the complexity of the relationship between sleep problems, emotional and behavioural difficulties, and autism symptoms. Depression and anxiety emerged in several analyses as highly connected symptoms and, as such, should be a focus for future research examining supports for autistic children, since targeting these areas may reduce the connectivity between symptoms and the subsequent risk of co-occurring conditions.

## Electronic supplementary material

Below is the link to the electronic supplementary material.

Supplementary Material 1

## Abbreviations

ASD: Autism spectrum disorder
RRBs: Restricted repetitive and stereotyped behaviour
