# Supplementary material for: The Connection Between Sleep Problems and Emotional and Behavioural Difficulties in Autistic Children: A Network Analysis
Source: J Autism Dev Disord. 2024 Mar 25;55(4):1159–71. doi: 10.1007/s10803-024-06298-2 (PMC11933199; doi:10.1007/s10803-024-06298-2)
Supplement: Supplementary file 1 — Supplementary Material 1 [file 10803_2024_6298_MOESM1_ESM.docx]

**Supplementary Tables**

Table S1. *Partial correlations between sleep problems, emotional and behavioural difficulties and autism symptoms in autistic children.*

|  | Emotional Behavioural Difficulties ^a^ | | | | |  | | Sleep Problems ^b^ | | | | |  | | Autism Symptoms ^c^ | | | |
| --- | --- | --- | --- | --- | --- | --- | --- | --- | --- | --- | --- | --- | --- | --- | --- | --- | --- | --- |
| Measure | Behavioural difficulties | Anxiety | Depression | Hyperactivity |  | | Sleep initiation & duration | | Sleep anxiety/co-sleeping | Night waking/ parasomnias | Daytime alertness |  | | Social interaction | | Communication | RRB’s |  |
| Behavioural difficulties | - |  |  |  |  | |  | |  |  |  |  | |  | |  |  |  |
| Anxiety | .00 | - |  |  |  | |  | |  |  |  |  | |  | |  |  |  |
| Depression | .47 | .43 | - |  |  | |  | |  |  |  |  | |  | |  |  |  |
| Hyperactivity | .65 | .00 | -.12 | - |  | |  | |  |  |  |  | |  | |  |  |  |
| Sleep initiation & duration | .00 | .00 | .18 | .00 |  | | - | |  |  |  |  | |  | |  |  |  |
| Sleep anxiety/co-sleeping | .00 | .28 | -.13 | .00 |  | | .16 | | - |  |  |  | |  | |  |  |  |
| Night waking/parasomnias | .00 | .36 | .00 | .00 |  | | .00 | | .20 | - |  |  | |  | |  |  |  |
| Daytime alertness | .00 | .00 | .31 | -.13 |  | | .00 | | -.13 | .00 | - |  | |  | |  |  |  |
| Social interaction | .00 | .00 | .00 | .00 |  | | .00 | | .00 | .00 | .00 |  | | - | |  |  |  |
| Communication | .00 | .00 | .00 | .00 |  | | .00 | | .00 | .00 | .00 |  | | .46 | | - |  |  |
| RRB’s | .00 | .14 | .00 | .12 |  | | .00 | | .00 | .00 | -.01 |  | | .12 | | .00 | - |  |

^a^ DBC ^b^ CSHQ-Autism ^c^ SCQ-C

Table S2. *Centrality Indices of Network*

| Node | Dimension | Strength |
| --- | --- | --- |
| Social interaction | 1 | 0.576 |
| Communication | 1 | 0.457 |
| Behavioural difficulties | 2 | **1.123** |
| Depression | 2 | **1.643** |
| Hyperactivity | 2 | 1.019 |
| Daytime alertness | 2 | 0.565 |
| Anxiety | 3 | **1.218** |
| Sleep initiation and duration | 3 | 0.346 |
| Sleep anxiety/co-sleeping | 3 | 0.910 |
| Night waking/parasomnias | 3 | 0.566 |
| RRBs | 3 | 0.385 |

*Note.* The three most central nodes based on strength index are reported in **bold.** Betweenness and closeness were not included as stability analysis was not stable, thus not interpretable. Only strength was reported, which reach the required cut-off of 0.5.

*Figure S1.* Network (CSHQ-Autism, SCQ-C and DBC) average correlation between centrality indices of networks sampled with persons dropped and the original sample. Line indicates the *mean* and *area* indicate the range from 2.5^th^ quantile to the 97.5^th^ quantile.
